# Long-term consequences of osteoporosis therapy with bisphosphonates

**DOI:** 10.20945/2359-4292-2022-0334

**Published:** 2023-11-10

**Authors:** Bárbara Gehrke, Maria Caroline Alves Coelho, Catarina Brasil d'Alva, Miguel Madeira

**Affiliations:** 1 Universidade Estadual do Rio de Janeiro Fisiopatologia Clínica e Experimental Rio de Janeiro RJ Brasil Programa de Pós-graduação em Fisiopatologia Clínica e Experimental (FISCLINEX), Universidade Estadual do Rio de Janeiro, Rio de Janeiro, RJ, Brasil; 2 Universidade Estadual do Rio de Janeiro Centro de Pesquisa Clínica Multiusuário Rio de Janeiro RJ Brasil Centro de Pesquisa Clínica Multiusuário (CePeM), Universidade Estadual do Rio de Janeiro, Rio de Janeiro, RJ, Brasil; 3 Universidade Estadual do Rio de Janeiro Faculdade de Ciências Médicas Departamento de Medicina Interna Rio de Janeiro RJ Brasil Divisão de Endocrinologia, Departamento de Medicina Interna, Faculdade de Ciências Médicas, Universidade Estadual do Rio de Janeiro, Rio de Janeiro, RJ, Brasil; 4 Universidade Federal do Ceará Núcleo de Atendimento Multidisciplinar às Doenças Osteometabólicas Departamento de Medicina Clínica Fortaleza CE Brasil Núcleo de Atendimento Multidisciplinar às Doenças Osteometabólicas, Departamento de Medicina Clínica, Universidade Federal do Ceará, Fortaleza, CE, Brasil; 5 Universidade Federal do Rio de Janeiro Divisão de Endocrinologia Rio de Janeiro RJ Brasil Divisão de Endocrinologia, Universidade Federal do Rio de Janeiro (UFRJ), Rio de Janeiro, RJ, Brasil

**Keywords:** Bisphosphonate, osteoporosis, fracture, osteonecrosis of the jaw, atypical femoral fracture

## Abstract

Bisphosphonates (BPs) are medications widely used in clinical practice to treat osteoporosis and reduce fragility fractures. Its beneficial effects on bone tissue have been consolidated in the literature for the last decades. They have a high affinity for bone hydroxyapatite crystals, and most bisphosphonates remain on the bone surface for a long period of time. Benefits of long-term use of BPs: Large and important trials (Fracture Intervention Trial Long-term Extension and Health Outcomes and Reduced Incidence with Zoledronic acid Once Yearly-Pivotal Fracture Trial) with extended use of alendronate (up to 10 years) and zoledronate (up to 6 years) evidenced significant gain of bone mineral density (BMD) and vertebral fracture risk reduction. Risks of long-term use of BPs: The extended use of antiresorptive therapy has drawn attention to two extremely rare, although severe, adverse events. That is, atypical femoral fracture and medication-related osteonecrosis of the jaw are more common in patients with high cumulative doses and longer duration of therapy. BPs have demonstrated safety and effectiveness throughout the years and evidenced increased BMD and reduced fracture risks, resulting in reduced morbimortality, and improved quality of life. These benefits overweight the risks of rare adverse events.

## INTRODUCTION

Bisphosphonates (BPs) are medications with consolidated evidence of anti-osteoporotic effect, and they have already been used for more than two decades in the clinical field (1). The benefits of fracture prevention and bone mineral density (BMD) increase are maintained for years during and after discontinuation of treatment with BP (1–5). Other positive aspects of these agents are lower healthcare costs, reduced morbidity, and significantly increased survival rates (6). Three-year use of zoledronic acid (ZOL) reduces mortality in individuals with low trauma hip fractures by 28% (6).

BPs are derivatives of inorganic pyrophosphate (PPi), which are chemically stable compounds. Studies accomplished in the 1960s evidenced that these compounds are able to hinder calcification by connecting to hydroxyapatite crystals. Therefore, the hypothesis that regulating PPi levels could control bone mineralization emerged (7). BPs differ from PPi in that a hydroxyl group is connected to the central carbon, which increases the ability to bind to calcium (7). The antiresorptive potency of BPs is determined by the presence of a nitrogen or amino group. These components increase the potency from 10 to 10.000 when comparing the first generation (clodronate and tiludronate) from the second and third generations (alendronate (ALN), risedronate (RIS), ibandronate (IBN), pamidronate, and ZOL) (7).

The Food and Drug Administration approved ALN in 1996, and since then, throughout several years, studies have been conducted to evaluate the efficacy and safety of BPs (1–5). ALN, RIS, and ZOL could reduce the risk of vertebral, nonvertebral, and hip fractures (1). IBN has been demonstrated to reduce the risk of vertebral fractures, and there is a paucity of data to prove the beneficial effect on hip and nonvertebral fractures (8,9).

BPs have been widely used in clinical practice to treat osteoporosis for several years. However, doubts concerning the duration of treatment and drug holidays remain (1). This article will review the beneficial effects and risks of long-term treatment with BPs.

## MECHANISM OF ACTION OF BPS

BPs are known to have an affinity for bone hydroxyapatite crystals and, therefore, bind to this component at the surface of the bone matrix. Moreover, BPs are incorporated into areas where bone remodeling is active (1,7,10,11). This group of drugs can prevent bone resorption and increase BMD and strength (10). The reduction of fracture in postmenopausal women, when compared to placebo, occurs after 1-2 years of treatment with BPs (1).

Those containing nitrogen in their composition act by binding and inhibiting the activity of farnesyl pyrophosphate synthase. This enzyme regulates the mevalonic acid pathway and plays an important role in producing cholesterol, other sterols, and lipids. The inhibition of farnesyl pyrophosphate synthase modifies the post-translational protein, ultimately leading to the inhibition of osteoclast recruitment and activity at the bone surface, as well as osteoclast apoptosis (7). BPs, therefore, inhibit the recruitment of osteoclasts and induce the apoptosis of these cells, resulting in the suppression of osteoclast-mediated bone resorption (1,10). They attach to the bone mineral surface owing to their high affinity to hydroxyapatite (Table 1), and the remaining dosage fraction is excreted by the kidneys (7,10,12–14). Patients with impaired renal function have decreased excretion of the medication; therefore, its use is not recommended in patients with a glomerular filtration rate of <30 mL/min (10). Moreover, they are hydrophilic and, therefore, have poor absorption in the gastrointestinal tract when administered orally (less than 1% absorption per oral dose) (7).

**Table 1:** Pharmacological characteristics of bisphosphonates considering affinity to hydroxyapatite and relative anti-resorptive potency. The table illustrates affinity to hydroxyapatite and relative anti-resorptive potency from lowest to highest, in order, from left to right

Characteristics	
Affinity	Risedronate < Ibandronate < Alendronate < Zoledronic acid
Relative anti-resorptive potency	Alendronate < Ibandronate < Risedronate < Zoledronic acid

RR: relative risk; CI: confidence interval; OR: odds ratio.

BPs remain in the bone structure for a long period of time (10). Pieces of evidence show that, when the bone that contains BP resorbs, a part of this medication is released and recirculates locally and systemically, thereby attaching once again to other surfaces and inhibiting bone resorption (15). This retention of BPs in the bone tissue explains the slow bone loss after therapy discontinuation as the drug may be retained for up to 10 years in the bone matrix (11,15).

## BENEFICIAL PIECES OF EVIDENCE FOR LONG-TERM USE OF BPS

Bone and cols. published in 2004 a multicenter study concerning the use of ALN in postmenopausal women for 10 years. Compared with baseline measurements, the ALN treatment resulted in a significant increase of BMD at the lumbar spine (13.7%), femoral neck (5.4%), and total hip (6.7%). The beneficial effect on BMD and bone remodeling markers was maintained, and the safety of the long-term therapy was confirmed. Nevertheless, a progressive loss of effect of the drug suspension was observed (5).

**Figure 1:**
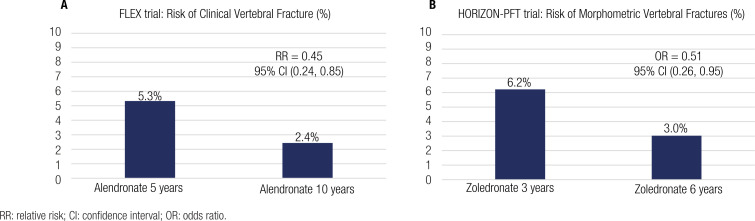
Vertebral fracture risk reduction in long-term use of bisphosphonates evidenced at FLEX and HORIZON-PFT trials. **A**. illustrates the smaller risk of clinical vertebral fractures at the group that used Alendronate for up to 10 years when compared to the group that used Alendronate for 5 years and switched to placebo for 5 years. **B**. illustrates the smaller risk of morphometric vertebral fractures at the group that used Zoledronate for up to 6 years when compared to 3 years of treatment with Zoledronate plus 3 years of placebo.

The Fracture Intervention Trial Long-term Extension (FLEX) is another multicentric study that evaluated the effects of extended treatment with oral BPs (10 years) compared with the 5-year use in 1099 postmenopausal women (15). Women participating in the FLEX trial had to have a T-score of −1.6 standard deviation (SD) or less but not necessarily be in the osteoporosis range. Women who discontinued ALN after 5 years of therapy and switched to placebo presented a statistically significant (P < 0.001) increase in serum levels of bone turnover markers when compared with those that followed receiving ALN therapy (raise of 28.1% for alkaline phosphatase specific for bone (BSAP), 55.6% for C-telopeptide of type 1 collagen (β-CTX), and 59.5% for N-propeptide of type 1 collagen (P1NP)), although these serum levels remained lower than pretreatment levels. Moreover, individuals treated with ALN for 5 years followed by placebo had a statistically significant (P < 0.001) decrease in BMD at the lumbar spine and total hip compared with the group that maintained ALN for 10 years. The BMD at the spine and total hip decreased by −3.7% and −2.4%, respectively, in 10 years. Furthermore, as an exploratory outcome, the study documented the incidence of fractures for up to 10 years. The risk of clinical vertebral fractures in those treated with ALN for 5 years and switched to placebo was over two times higher than those using 5-10 mg daily of ALN for up to 10 years (5.3% versus 2.4%, respectively) (Figure 1A). Therefore, the relative risk reduction was 55%, and the absolute risk reduction was 2.9%. Concerning all clinical and nonvertebral fractures, no significant differences were documented. There was no increase in risk of nonvertebral fractures, and the risk of clinical vertebral fractures in the group treated with ALN for 10 years decreased compared to the group that switched to placebo at year 5. This result indicates that extended therapy (10 years) does not implicate adverse effects on bone strength. The high-risk groups comprise those with larger benefits in preventing vertebral clinical fractures: women with pre-existing fractures or those with very low BMD (T-score of −2.5 SD or less) at the baseline. The number needed for extended 10-year therapy is 25 in high-risk groups, compared with 50-300 in lower-risk groups (with no previous vertebral fracture or osteoporosis diagnosis) (1). Data from a post hoc subgroup analysis showed that, for women without vertebral fracture but with an osteoporotic range (T-score of −2.5 SD or less) at the femoral neck at the FLEX baseline, extended therapy for up to 10 years with ALN decreased the risk of nonvertebral fractures (3). The results of the FLEX study call attention to the fact that a 5-year therapy with BP has a long-term effect on bone tissue. The reason is that, based on observational studies, the cumulative effect on the total hip BMD was a 1%-3% gain compared with the 5%–10% loss in untreated women of the same age (16).

The Health Outcomes and Reduced Incidence with Zoledronic acid Once Yearly-Pivotal Fracture Trial (HORIZON-PFT) studied the long-term effects of ZOL 5 mg. A total of 1,233 osteoporotic postmenopausal women using ZOL for 3 years were randomized into two separate groups: placebo (n = 617) and 3 more years of ZOL (n = 616). The main endpoint analyzed was femoral neck BMD, and the other secondary endpoints were fractures, other BMDs, bone turnover markers, and safety issues. Bone turnover markers elevated in a 3-year ZOL group followed by 3 years of a placebo, whereas in the 6-year ZOL group, they remained steady although both presented smaller values than the baseline. Regarding bone turnover markers, P1NP increased in both groups with a 14% difference between them (P = 0.0001), and values remained under pretreatment. The 3-year ZOL group followed by placebo resulted in a statistically significant (P < 0.0009) inferior BMD at the femoral neck compared with the 6-year ZOL group, with a difference of 1.04%, although it still remained above baseline values. At the lumbar spine, the difference in BMD was 2.03% higher in the 6-year ZOL group compared with the 3-year ZOL group (P = 0.002). The risk of morphometric vertebral fractures in women treated with ZOL for 3 years and switched to placebo was approximately two times higher when compared with those using 5 mg yearly of ZOL for up to 6 years (6.2% versus 3.0%, respectively, odds ratio of 0.51 with P = 0.035) (Figure 1B). Regarding the incidence of all clinical fractures, nonvertebral fractures, and clinical vertebral fractures, no significant differences were documented, although confidence intervals were vast, and thus, we cannot rule out a possible benefit. Based on the results of this trial, we may conclude that postmenopausal women diagnosed with osteoporosis and high risk of fractures (particularly vertebral fractures) may benefit from continuous treatment with 5 mg yearly of ZOL for up to 6 years (2). A second extension of the HORIZON-PFT trial observed the results of 9-year extension therapy with ZOL. Women were randomized to a 3-year placebo after a 6-year therapy with ZOL or maintenance of treatment for up to 9 years with ZOL. No significant differences were observed in BMD values, bone turnover markers, and the number of fractures. Therefore, the results suggest that the extended treatment with ZOL for up to 9 years has no additional benefit, although the sample size evaluated was much smaller than the previous extension study (190 women) owing to follow-up loss (17).

Furthermore, a smaller study with extended use of RIS published in 2004 observed the effects of a 7-year treatment in postmenopausal women with osteoporosis. The endpoints were BMD analysis, bone turnover marker levels, and assessment of vertebral, and nonvertebral fractures. The groups included 5 years of placebo followed by 2 years of RIS (n = 81) and 7 years of RIS group (n = 83); all 136 women completed the study. The study evidenced an increase in BMD measurements after 5 years of treatment with RIS when compared with the baseline and remained stable or increased in patients treated with 7 years of RIS. BMD at the lumbar spine raised 8.8% in the 5-year RIS group and 11.5% in the 7-year RIS group when compared with the study onset. Considering bone turnover markers, in the 7-year RIS group, N-telopeptide measured in urine decreased by 54% at 3 months and 63% at 7 years, when compared with the baseline. The incidence of vertebral fractures was similar in groups using RIS for 5 years, suspended therapy, and RIS for up to 7 years, although a significant reduction in vertebral fractures was observed in the 5-year placebo group that began treatment with RIS for 2 years. The incidence of nonvertebral fractures in a 5-year placebo followed by 2-year RIS was 7.4%, whereas the 7-year RIS group presented a lower incidence of 6.0% but was not significant. Further analysis demonstrated that once patients started receiving treatment with RIS after the 5-year placebo therapy, fracture incidence was diminished. The reason is that there was a statistically significant reduction of new fractures when compared with 4-5 years of placebo (2 *vs.* 12 patients, with P = 0.008). The study concluded that extended use of RIS maintains anti-fracture efficacy and is well tolerated for a long period of time (18).

Therefore, the task force of the American Society for Bone and Mineral Research (ASBMR) reaffirms the results and conclusions of the studies described above. The ASBMR also suggests that postmenopausal women receiving oral BP for ≥ 5 years or intravenous BP for ≥ 3 years and with hip, spine, or multiple other osteoporotic fractures before or during therapy or hip BMD T-score of ≤ −2.5 SD or high risks of fracture (obtained using fracture risk calculators) will benefit from maintaining BP or switching to another anti-fracture therapy and should be reassessed every 2-3 years (19).

## RISKS ASSOCIATED WITH THE USE OF BPS

Adverse events non-related to extended use of BP are gastrointestinal symptoms with oral BPs, flu-like symptoms when administered intravenously, hypocalcemia, musculoskeletal pain, and atrial fibrillation (Table 2) (7,11). Individuals with known gastroesophageal reflux disease or with esophageal stricture are most affected by oral BP administration owing to esophageal irritation and erosion (7). Concerning flu-like symptoms, approximately 10%-30% of individuals that received the first infusion of nitrogen-containing BP present an acute phase reaction marked by influenza-like symptoms (headache, arthralgia, myalgia in association with transient pyrexia) (7). Moreover, hypocalcemia may occur after intravenous BP infusion, most frequently in patients with high rates of bone resorption mediated by osteoclasts, renal dysfunction, hypovitaminosis D, or undiagnosed cases of hypoparathyroidism (7). Severe musculoskeletal pain is a rare adverse event that may occur at any phase of intravenous or oral BP treatment (7).

**Table 2:** Bisphosphonates adverse events and duration of therapy

Short-term adverse events	Long-term adverse events
Gastrointestinal symptoms (oral administration)	Medication-related osteonecrosis of the jaw
Hypocalcemia	Atypical femoral fracture
Musculoskeletal pain	
Atrial fibrillation	
Flu-like symptoms (intravenous administration)	

Approximately 10 years after BP approved the osteoporosis treatment, post-marketing reports based on the long-term treatment of millions of patients revealed the occurrence of two initially unexpected adverse events: atypical femoral fracture (AFF) and medication-related osteonecrosis of the jaw (MRONJ). Both conditions are extremely rare and, therefore, must not hinder the long-term use of these antiresorptive agents when indicated (6).

Concerns regarding the long-term use of BP draw attention to the possibility that normal physiological bone turnover and repair may be reduced. This case may lead to the accumulation of microdamage or microcracks and reduced bone strength leading to AFF (1,15). Table 3 presents the major and minor criteria of AFF (6,20). The locations where AFF occur are considered atypical as they are regarded as the most resistant region of the femur, thereby being improbable to fracture with low trauma in the absence of fragility (1). Subtrochanteric and diaphyseal fractures account for 7%-10% of all femoral fractures, and among them, 75% are related to major trauma (20). Other risk factors for the occurrence of AFF are low 25-hydroxyvitamin D concentrations (less than 20 ng/mL), diabetes, use of a glucocorticoid, low total hip BMD, history of falls, older age, and femoral shape (20,21).

**Table 3:** Minor and Major criteria of atypical femoral fractures (AFF). Four of the five major criteria must be present for the diagnosis of AFF

Minor criteria:	Major criteria:
Use of medications or comorbidities that increase risk of AFF Symptoms of groin or thigh pain Bilateral fractures Increase in cortical thickness of diaphysis Lateral cortex periosteal reaction Delayed healing	Spontaneous or minimal trauma fracture Absence of comminution Located at femoral shaft or subtrochanteric region Short oblique or transverse orientation Incomplete fractures involving lateral cortex, while complete fractures extend through both cortices

Commonly, AFF begins with a periosteal callus shown as a blurred formation, posteriorly becoming solidified. The periosteal calluses reflect an attempt to cure bone tissue before evolving into a fracture, and they occur in AFF near the developing fracture on the lateral cortex of the bone (20). Patients with this complication may present contralateral fracture in 28%, healing delay in 26%, and thigh pain in 30%-70% of cases (22). Therefore, whenever the patient with extended use of BP complains of groin or thigh pain, an urgent bilateral femoral imaging evaluation is required to detect partial fracture, stress reaction, or total hip fracture (20). Imaging may be used to diagnose AFF, and conventional radiography seems convenient, although advanced imaging technologies (scintigraphy, computerized tomography scan, and magnetic resonance imaging) present higher specificity and sensitivity for detection in the initial phases (20).

The absolute risk of AFF in patients undergoing BP treatment ranges from 3.2 to 50 cases per 100.000 person-years with short-term use (<5 years) and approximately 113 per 100.000 person-years with long-term use (>5 years) (23). A recently published study evidenced that, in women treated with BP for over 3 years, the 5-year, and 10-year cumulative incidences of AFF were above fourfold and 10-fold, respectively, compared with women treated with BP for less than 3 years (24). Schilcher and cols. carried out a Swedish study and observed that for each additional year of BP use, the adjusted odds of AFF increase by 2.5-fold (25). However, although the relative risk of AFF is higher in patients on BP treatment, the absolute risk is uniformly very low among studies (20).

The risk of AFF should be analyzed alongside the benefits of preventing osteoporotic fractures. The use of amino-BPs in high-risk patients (with clinical vertebral fractures) is estimated to avoid 3300 clinical fractures per 100.000 person-years of treatment, whereas its use in moderate-risk patients (femoral neck BMD T-score < −2.0) prevents 1,700 clinical fractures per 100.000 person-years (20,26). Moreover, for each 100 typical femoral neck or intertrochanteric fractures prevented by BP use, one subtrochanteric atypical fracture occurs (27). Therefore, we may conclude that the risk-benefit analysis favors BP therapy for women at high risk for fracture.

MRONJ is an uncommon (prevalence varies from 0% to 0.04%) but threatening condition described in patients undergoing antiresorptive treatment with BP (11). This condition occurs in approximately 1.04-69 cases per 100.000 person-years. Patients with diagnosed cancer associated with malignant hypercalcemia or bone metastasis present a significantly higher incidence of MRONJ, ranging from 1% to 10% (28). This complication is defined as the “presence of exposed jaw bone, or bone that can be probed, through an intraoral or extraoral fistula, for at least 8 weeks in patient with history of antiresorptive and/or antiangiogenic therapy, and in the absence of previous radiation therapy to head and neck”. The diagnosis of MRONJ is clinical, although imaging may be accomplished to assist in doubtful cases (11).

Similar to AFF, the duration of antiresorptive treatment, including adherence to therapy, has been identified as a risk factor for MRONJ (11,29–31). Other risk factors are a history of cancer, chronic use of glucocorticoids (or antiangiogenic, antithrombotic, immunosuppressants, proton pump inhibitors, or antiresorptive agents), smoking, hypertension, rheumatoid arthritis, or *diabetes mellitus*. In addition, local risk factors may contribute to the development of this adverse event, such as invasive oral procedures, trauma, poor oral hygiene, inflammatory oral diseases, and dental infection (11).

A recently published position paper reunites the recommendations of three important societies: the Brazilian Society of Endocrinology and Metabolism (SBEM), the Brazilian Society of Stomatology and Oral Pathology (SOBEP), and the Brazilian Association for Bone Evaluation and Osteometabolism (ABRASSO). The paper recommends that patients considered to initiate therapy with an antiresorptive agent must be referred to dentists for prior orientation and preventive procedures (11). Moreover, no evidence supports the interruption of BP treatment prior to dental procedures to inhibit MRONJ (11,32), including the use of β-CTX serum levels to predict the risk of MRONJ before oral procedures (11). Regular preventive dental evaluations, orientations for oral hygiene, and prophylactic use of antibiotics before procedures, such as extraction, should be considered. Moreover, plain amoxicillin or its association with clavulanate is highly recommended (11). For those who are allergic to penicillin, clindamycin may be used. The literature described several prophylactic protocols regarding the period of use of antibiotics. Data suggest initiating therapy 48-72 hours prior to the dental procedure and maintaining treatment for 1-3 weeks after the procedure (11).

Patients that need to maintain anti-osteoporotic treatment owing to the high risk of fracture may use anabolic treatment to enhance regeneration of MRONJ (improvement of bone regeneration ratio and bone turnover markers), according to data described in the literature (33,34). In addition, more favorable outcomes were described when recombinant human bone morphogenetic protein 2 (rhBMP-2) was used in association with teriparatide, suggesting that rhBMP-2 boosts bone regeneration in MRONJ cases (33). Similarly, patients suffering from AFF may consider using teriparatide as an adjunct therapy, particularly those with no evidence of healing after 4-6 weeks following the surgical procedure (20,35). The literature described few cases of healing improvement owing to the anabolic effect of the drug (20). After 2 years of treatment with an anabolic agent, antiresorptive therapy may be resumed and must be individualized, considering the risk of fracture, resolution of adverse event (MRONJ or AFF), and type of treatment (surgical or not). Anti-osteoporotic agents that may be used are raloxifene, denosumab, BPs, and hormone replacement therapy (36).

In conclusion, through this review of the benefits and risks of the extended use of BP, we may conclude that the effects on BMD and the risk of fracture observed in long-term treatment far outweigh the risks of rare and severe adverse events. These positive effects are not restricted to bone changes once there is a reduction of morbimortality and an increase in patients’ quality of life who receive adequate treatment for osteoporosis. Extended therapy with BP should be considered in patients with a high risk of fragility fractures (2,6,15,22). Thus, the decision to continue or interrupt therapy with BPs is a challenge and should follow the recommendations described above.
